# Algorithm-based mapping of products in a branded Canadian food and beverage database to their equivalents in Health Canada’s Canadian Nutrient File

**DOI:** 10.3389/fnut.2022.1013516

**Published:** 2023-02-16

**Authors:** Sappho Z. Gilbert, Conor L. Morrison, Qiuyu J. Chen, Jesman Punian, Jodi T. Bernstein, Mahsa Jessri

**Affiliations:** ^1^Department of Chronic Disease Epidemiology, Yale School of Public Health, New Haven, CT, United States; ^2^Food, Nutrition, and Health Program, Faculty of Land and Food Systems, The University of British Columbia, Vancouver, BC, Canada; ^3^Department of Statistics, Faculty of Science, The University of British Columbia, Vancouver, BC, Canada; ^4^Centre for Health Services and Policy Research (CHSPR) and Health Services and Policy (HSP), Faculty of Medicine, The University of British Columbia, Vancouver, BC, Canada

**Keywords:** database mapping, nutritional surveillance and monitoring, food composition tables (FCTs), food supply, public health nutrition, fuzzy matching, Canada, Canadian Nutrient File

## Abstract

**Introduction:**

There is increasing recognition of the value of linking food sales databases to national food composition tables for population nutrition research.

**Objectives:**

Expanding upon automated and manual database mapping approaches in the literature, our aim was to match 1,179 food products in the Canadian data subset of Euromonitor International’s Passport Nutrition to their closest respective equivalents in Health Canada’s Canadian Nutrient File (CNF).

**Methods:**

Matching took place in two major steps. First, an algorithm based on thresholds of maximal nutrient difference (between Euromonitor and CNF foods) and fuzzy matching was executed to offer match options. If a nutritionally appropriate match was available among the algorithm suggestions, it was selected. When the suggested set contained no nutritionally sound matches, the Euromonitor product was instead manually matched to a CNF food or deemed unmatchable, with the unique addition of expert validation to maximize meticulousness in matching. Both steps were independently performed by at least two team members with dietetics expertise.

**Results:**

Of 1,111 Euromonitor products run through the algorithm, an accurate CNF match was offered for 65% of them; missing or zero-calorie data precluded 68 products from being run in the algorithm. Products with 2 or more algorithm-suggested CNF matches had higher match accuracy than those with one (71 vs. 50%, respectively). Overall, inter-rater agreement (reliability) rates were robust for matches chosen among algorithm options (51%) and even higher regarding whether manual selection would be required (71%); among manually selected CNF matches, reliability was 33%. Ultimately, 1,152 (98%) Euromonitor products were matched to a CNF equivalent.

**Conclusion:**

Our reported matching process successfully bridged a food sales database’s products to their respective CNF matches for use in future nutritional epidemiological studies of branded foods sold in Canada. Our team’s novel utilization of dietetics expertise aided in match validation at both steps, ensuring rigor and quality of resulting match selections.

## 1. Introduction

In recent decades, food marketing and retail databases have been revisited as a largely untapped source of low-bias, high-quality data for researching trends in consumer health and nutrition (1). Such databases can also be utilized for the study of front-of-pack labeling, marketing and advertising to children, and the implementation and surveillance of dietary guidelines (2). For food and beverage manufacturing industries, these data on the nutrient content of their products and associated sales can guide healthy eating initiatives and even product reformulations (3). When used with food composition information, these data can be especially useful for health professionals and scholars, including clinicians, dietitians, and epidemiologists studying the impact of population diet and nutrition on the prevalence and incidence of certain diet-related chronic conditions (4). Innovative approaches to monitoring public health and community nutrition are particularly critical and can be enabled using these kinds of datasets (1, 5).

Researchers are increasingly seeing the value for public health nutrition and nutritional epidemiology in linking food retail, manufacturing, and marketing datasets to food composition tables. Digital data on food sold in stores–such as *via* electronic point-of-sale systems–commonly include information on the quantities sold, price paid, and promotion status (4–7). These data are ubiquitous in the food retail industry and are already utilized in product development and marketing (4).

Marketing analytics databases that track trends in food sales (including for branded products) are similarly enticing for their potential use as longitudinal observation data and in scenario modeling for food policy and public health nutrition interventions; examples include multinational marketing analytics companies like Euromonitor, Kantar Worldpanel, GlobalData, Nielsen, and Gesellschaft für Konsumforschung (GfK) (8). However, realizing the potential of their sales data for population nutrition research and policy depends on the availability of corresponding food composition data. Some databases provide extensive nutrient data for their products. Nielsen (through a sister company, Brand Bank) and Kantar have nutrient data available for their tracked products (8). Meanwhile, in cases where these databases only have partial food composition data (like Euromonitor, which provides product information for energy and 7 nutrients) or none at all (such as GfK or Global Data), matching to a national, commercial, or other food composition database is likely needed (1, 8).

These marketing companies’ data have recently been used to evaluate health-related interventions in diverse contexts across the globe, such as Denmark’s saturated fat tax and nutrition assistance programs in the United States (1). These databases may also be used to overcome limitations of national food composition tables such as the Canadian Nutrient File (CNF). The CNF forms the basis for national health surveys in Canada and contains mostly generic aggregates of foods, is not systematically or consistently updated, and is largely based on data from the United States Department of Agriculture (USDA) (9, 10). Since some of the aforementioned companies include data on food composition of specific branded foods and beverages, their data could be a beneficial supplement to the CNF for use in population dietary surveillance. Even for the exact foods for which the brand is known, CNF information on these branded products may be outdated and not reflective of the current Canadian food supply. Additionally, many CNF foods are generic estimates of products available on the market, with some being the average of many types of that food; while this can be a boon to those seeking a more complete nutrient profile and representative nutrient data for a non-brand-specific food, it is conversely a limitation for those focused on specific branded products.

Prior studies have reported various approaches to linking food retail and marketing datasets to food group and composition data for the study of population health and nutrition, including both manual and algorithm-based approaches (1, 4, 5, 6). The traditional way of mapping a database of products to their respective food composition (nutrient content) is by manual matching; generally, this means a product is matched to a food or beverage item code in a food composition database, which then pulls in those nutrient data for use with that now-linked product (8). This can be very resource-intensive and error-prone, as it tends to require significant amounts of time and effort to match food items and/or categories manually. Brinkerhoff et al. (4) attempted automated mapping of foods in a supermarket dataset to their nutritional equivalents in the USDA’s Standard Reference (USDA-SR) database but found a relatively small number of successful matches due to differences in food naming strategies and categorization conventions; as a result, manual matching was performed in full.

As long as new products enter the food supply, matching will remain a data maintenance problem–a reality that underscores the need for efficient, replicable, and adaptable matching protocols. An effective algorithm that can (at least) partially automate matching of food products to their closest equivalent in food composition tables would alleviate some of these manual matching burdens (especially in large databases). Other scholars have been able to create algorithms for foods across databases that appear to lead to good matches (8, 11, 12). However, most such articles do not report expert validation of these matches for their compositional closeness. The aim of this work was thus to design a primarily automated, dietetics expert-validated methodology for matching food and beverage products in the Euromonitor Passport Nutrition’s Canada subset to their equivalents in the CNF.

## 2. Methods

Products in the Canada-wide Euromonitor data subset (“Euromonitor database”) were linked with those in the Canadian Nutrient File (CNF) using the following methodology (10). Our Bureau of Nutritional Sciences (BNS)-integrated Nutrition and Fuzzy Match (BiNFM) algorithm was coded in R (13). Fuzzy string matching refers to a class of algorithms designed to determine the similarity of two unequal strings. In our case, we used the “partial token sort ratio” fuzzy matching algorithm that is implemented by the fuzzywuzzyR package in R (14), which ports the fuzzywuzzy package from Python (15). This particular fuzzy matching algorithm takes two strings as input and then outputs a score from 0 (indicating no similarity) to 100 (indicating near exact similarity).

Two research groups have developed algorithms to automate a similar database mapping process and reported their methods and reflections (11, 12). Elements of both of their approaches were found to be applicable to our work and were adapted to fit the nature and challenges of our matching effort. In short, motivated by Tran et al., we restricted our algorithm to only suggest matches where the Euromonitor product and the CNF food(s) shared a food category in common; inspired by Lamarine et al., we also employed fuzzy string matching in our algorithm (11, 12). Divergences from these previous works included our use of nutrient-based thresholds in the algorithm and the addition of the dietetics expert validation of final match selections, which are described in greater detail below.

### 2.1. Overview of databases

#### 2.1.1. Euromonitor data subset of branded Canadian consumer food products sold between 2014 and 2018

**FIGURE 1:**
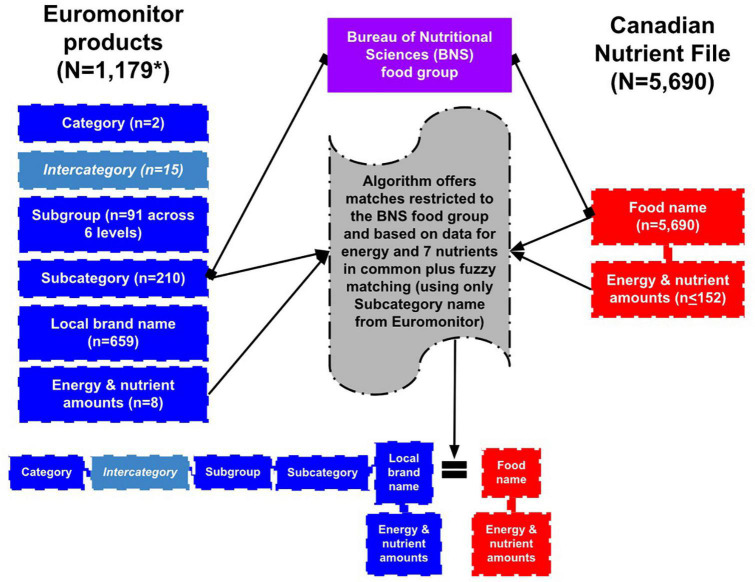
Overview of the architectures of Euromonitor’s Canadian data subset in Passport Nutrition and the Canadian Nutrient File (CNF), with an emphasis on the key variables used in their database mapping. *Of the 1,179 total Euromonitor products, only 1,111 were ultimately able to be run through the algorithm.

Euromonitor International Ltd. (London, UK) is a market research company whose Passport Nutrition database offers nutrition data for products sold in different countries worldwide. We acquired a subset of this data that contained major branded foods and beverages sold in Canada from 2014 to 2018. As visualized in Figure 1, this dataset included 1,179 products from two main categories (Packaged Food and Soft Drinks) across 210 subcategories. A single Euromonitor product consists of two parts: (1) the subcategory it belongs to, and (2) the brand name. For example, “Children’s Breakfast Cereals” (subcategory) + “President’s Choice” (brand) = “Children’s Breakfast Cereals, President’s Choice” (= 1 Euromonitor product). Euromonitor provides definitions for each subcategory that outline the types of foods and leading market brands. For each product, data for energy and 7 nutrients are reported: carbohydrate, protein, total dietary fat, saturated fat, sugar, fiber, and salt (which was converted to sodium: grams of salt x 393 = milligrams of sodium).

#### 2.1.2. Canadian Nutrient File (CNF)

The CNF is a national food composition database. Its latest version from 2015 was used in this work and contains 5,690 Canadian foods and data for up to 152 nutrients for each (10). The majority of CNF food names are presented as generic food descriptions (e.g., “Cheese, blue”), with a minority of foods containing brand-specific information in their names (e.g., “Cereal, ready to eat, Cheerios, General Mills”). CNF foods exist within 23 broadly named food categories (e.g., Dairy and Egg Products, Breakfast Cereals, and Nuts and Seeds).

#### 2.1.3. Bureau of Nutritional Sciences (BNS) food groups

Bureau of Nutritional Sciences is a food category system that contains a granular classification scheme developed by Health Canada for categorizing foods (13). Due to the variability in food categorization between the Euromonitor database and the CNF, BNS food groups were utilized as a bridging tool between these two databases. Each Euromonitor product and CNF food had a BNS food group assigned to them manually by dietetics experts; while there are 78 such food groups in the BNS, only 50 were used in this project (as 28 were excluded for being dishes rather than individual foods). To optimize its efficiency and accuracy, our algorithm was designed to only offer potential matches between Euromonitor products and CNF foods that share the same BNS food group.

### 2.2. BiNFM algorithm design

Our algorithm’s matching relies on the names of Euromonitor subcategories and CNF foods, BNS food groups shared in common, and the differences in the nutrients of Euromonitor products and CNF foods. Figure 1 visually depicts our database mapping approach as it relates to algorithm design, which is described step-by-step below.

Given a particular Euromonitor product, the algorithm sifts (in rounds that we fittingly also term “sifts”) through all potential CNF foods to produce a list of suggested CNF matches. Initially, this list consists of all CNF foods that share a BNS group with the Euromonitor product. The sifting process then applies up to five filters to this list of potential matches to arrive at the algorithm-suggested match options. Two of these filtering steps are marked as optional, as they were only employed for a subset of sifts in our study. Including these optional filters will generally provide a narrower, plausibly more specific list of suggested matches but may result in a lower sensitivity. The cost-benefit analyses of which optional filters to use will vary on a case-by-case basis. The five filtering steps are:

1. Only CNF foods that have macronutrient (carbohydrate, protein, or total fat) contents that differ from the Euromonitor product by an amount falling below a predefined threshold are kept as potential matches. The difference in the content of a nutrient X as a proportion of calories between foods in Euromonitor (E) and in the CNF (C) is equal to = (Nutrient X as a % of Calories in E) – (Nutrient X as a % of Calories in C). For example, if 30% of the calories in a given Euromonitor product are from carbohydrates, and 20% of the calories in a given CNF food are from carbohydrates, then the difference in carbohydrates as a proportion of calories is 10%. The thresholds we used are described later in this subsection.

Differences in nutrients as a proportion of calories are used to better account for differences in nutrient contents for foods across a large range of total caloric contents. This approach was found to be more robust than either absolute or relative differences in the grams of nutrients when simultaneously wanting to compare nutrient contents of high-calorie and very low-calorie items. As an example of absolute differences (in grams of nutrient) being less robust, consider a difference of 5 g in carbohydrates. A 5 g difference might be small for a product with 100 g of carbohydrates, but this is large for one with only 10 g of total carbohydrates. Using relative differences is non-robust for low-calorie items. For example, a product with only 1 g total carbohydrate that faces a 1 g difference with another food will equal a 100% difference—even though the absolute difference is only a mere 1 g per 100 g of product. Differences as a proportion of calories were thereby found to be more useful for both low and high nutrient foods, as the nutrients in each product are normalized by their energy content.

2. Only CNF foods that have fiber, saturated fat, and sugar contents as a proportion of calories that differ from the Euromonitor product by an amount falling below a predefined threshold are kept as potential matches.

3. If the Euromonitor product has a non-zero sodium content, the relative difference in sodium content is computed between the Euromonitor product and the CNF food and this quantity is compared to a third threshold. The relative difference in the sodium content of Euromonitor (E) vs. CNF (C) is computed as = (Sodium content of E in mg – Sodium content of C in mg) ÷ (Sodium content of E in mg).

4. (*Optional; only used in 1 of our 4 sifts*) Only CNF foods whose fuzzy match score with the Euromonitor product exceed a set threshold are kept as potential matches.

5. (*Optional; used in 3 of our 4 sifts*) Only CNF foods whose fuzzy match score with the Euromonitor product are equal to the largest fuzzy match score between the product and all potential CNF matches remaining from the previous filtering step are kept as potential matches.

Henceforth we will refer to the chosen thresholds and optional steps as “sift parameters,” or more leniently as “parameters.” To ensure that there were matches suggested for each Euromonitor product, several sifts were run with a variety of parameters. If a Euromonitor item had fewer suggested matches than desired, it was included in a subsequent sift with more lenient sift parameters. The parameters for each of the run sifts are detailed in Table 1. For example, in the *“First”* sift, the difference threshold for protein, carbohydrates, and total dietary fat as proportions of calories was set to 20%; for fiber, saturated fat, and sugar as proportions of calories was set to 10%; and for the relative difference threshold for sodium contents was set to 50%. An additional sift, “*First*+,” used more lenient matching parameters, and was applied to the Euromonitor products which had single CNF matches from “*First*.” This was done to increase the number of potential CNF matches and the overall sensitivity of the algorithm.

**TABLE 1:** Sets of thresholds as differences between Euromonitor and Canadian Nutrient File (CNF), as applied in each sift of our algorithm.

Sift			*First*	*First*+	*Second*	*Third*
**Total number of Euromonitor products run through sift**			1111	591	207	43
Maximum difference^a^	*All 3 conditions must be simultaneously met:*	Total dietary fat	20%	60%	40%	∞
		Carbohydrate	20%	60%	40%	∞
		Protein	20%	60%	40%	∞
	*All 3 conditions must be simultaneously met:*	Fiber	10%	60%	40%	∞
		Saturated fat	10%	60%	40%	∞
		Sugar	10%	60%	40%	∞
	Sodium		50%	50%	50%	∞
Minimum fuzzy matching score (0–100)^b^			0	50	0	0
Fuzzy match optimization used^c^			Yes	No	Yes	Yes

∞Indicates no maximum difference.

^a^Maximal difference thresholds for all nutrients except sodium were based on differences in those nutrients as a proportion of calories, while the maximal threshold for sodium was based on relative differences.

^b^The fuzzy matching score system was a continuum between 0 and 100 (inclusive).

^c^Fuzzy match optimization was applied to select one or more CNF foods with the highest fuzzy matching score out of the list of potential CNF matches for each Euromonitor product.

**FIGURE 2:**
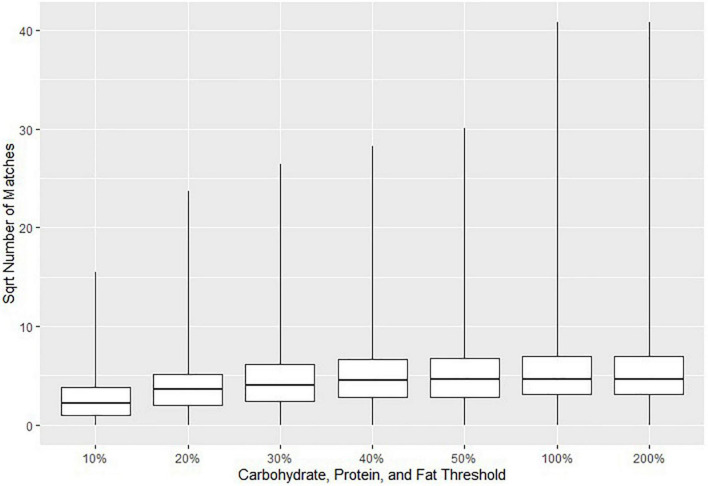
Boxplots indicating the 0th, 25th, 50th, 75th, and 100th percentiles of the square roots of the numbers of Canadian Nutrient File (CNF) suggested matches for all Euromonitor products as a function of the threshold selection for carbohydrates, proteins, and fats. The threshold for fiber, saturated fat, and sugar was set to 40% in all cases, and the sodium threshold was set to 50%. Fuzzy string matching was not used. The square root number of matches is reported due to the large numbers of matches when using higher thresholds.

**FIGURE 3:**
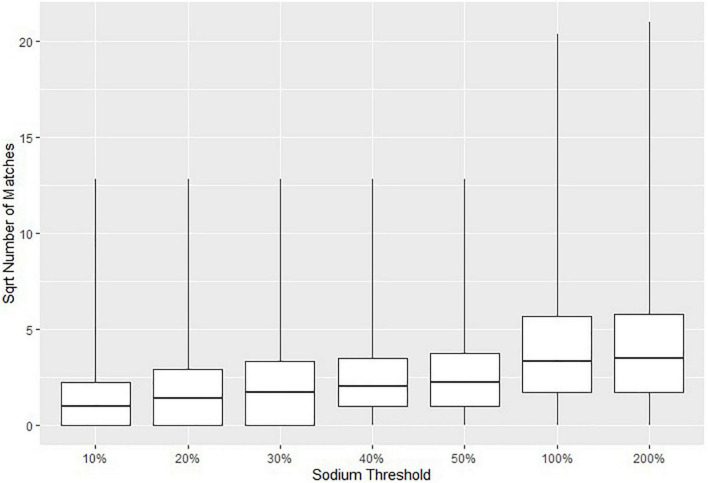
Boxplots indicating the 0th, 25th, 50th, 75th, and 100th percentiles of the square roots of the numbers of Canadian Nutrient File (CNF) suggested matches for all Euromonitor products as a function of the threshold selection for sodium. The threshold for carbohydrate, protein, and fats was set to 20% and the threshold for fiber, saturated fat, and sugar was set to 10% in all cases. Fuzzy string matching was not used. The square root number of matches is reported due to the large numbers of matches when using higher thresholds.

The parameters in Table 1 were selected based on two approaches. The first approach was to minimize the possible error in suggested matches to that of the assigned threshold (e.g., at most a 20% difference in macronutrient content). The second approach was to run the matching algorithm several times with different parameters to obtain a sufficient number of suggested CNF matches for each Euromonitor product. In general, higher thresholds would result in a greater number of suggested matches, but at the cost of a diminished specificity. Figure 2 visually demonstrates this by plotting the square roots (for easier readability) of the numbers of matches for all Euromonitor items at several candidate thresholds. Based on Figure 2, we heuristically decided that the difference in the number of matches suggested was most consequential when we changed the threshold for carbohydrates, proteins, and fats from 10 to 20% as well as from 50 to 100% (with thresholds for fiber, saturated fat, and sugar set to 40% and the sodium threshold set to 50%). This decision was based primarily on comparing the size of the differences in the median number and maximum number of matches for each of these thresholds. Figure 3 shows a plot demonstrating potential sodium thresholds when the thresholds for carbohydrate, protein, and fat were set to 20% and the thresholds for fiber, saturated fat, and sugar were set to 10%. Using a sodium threshold of 50% provided more matches than smaller thresholds, and a sodium threshold of 100% greatly increased the number of matches. With a similar reasoning as before, we used Figure 3 as a motivation for our choice of sodium threshold in the matching algorithm. This approach of parameter selection is a combination of numerical heuristics and nutrition expert judgment calls.

#### 2.2.1. Fuzzy matching in “First+”

*“First*+” used more lenient matching parameters and was applied to the 591 Euromonitor products with only one CNF match from “*First*.” In “*First*+,” a minimum fuzzy match score of 50 (out of 100 inclusive) was required for a CNF food to be considered a potential match–in addition to satisfying the nutrient thresholds (per Table 1).

#### 2.2.2. Selection among algorithm-proposed matches

All algorithm-proposed matches were nutritionally appropriate within an *a priori* error tolerance as specified by the aforementioned nutrient thresholds. Therefore, the dietetic validation of match selection among these options focused largely on the Euromonitor product’s qualitative data–namely, its subcategory (including definitions) and brand–in tandem with the name(s) of CNF food(s) suggested by the algorithm. In this way, the matching algorithm acts somewhat like an advanced search engine, whereby the results of the algorithm present an expert with a narrowed list of candidates for selection. Each of the candidates for matching already meets specified nutritional criteria, which frees time for the validator to focus on the features of the Euromonitor data that cannot be so easily understood solely by a computer.

**FIGURE 4:**
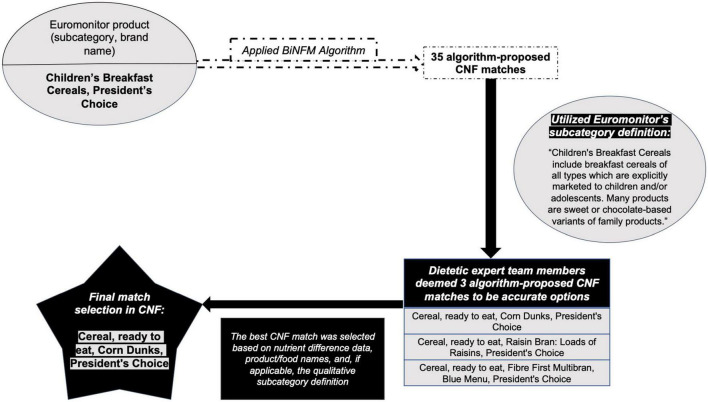
Example of our algorithm-aided, dietetics expert-validated matching procedure.

When the algorithm suggested at least one match for a given Euromonitor product, a dietetics expert team member would either choose the most accurate option (or it, if only one match was offered) or reject all suggested matches, thus sending that product for manual CNF selection. If the team member determined that multiple algorithm-proposed matches could be accurate, then the algorithm-proposed match that was deemed to have the least egregious nutritional error was selected as the most accurate (and final) match. To do this, the nutritional differences from steps 1, 2, and 3 of the algorithm (from Section “2.2 BiNFM algorithm design”) between the Euromonitor product and each suggested CNF food item were tabulated. Then, for each Euromonitor and CNF combination separately, the largest of these nutritional differences was computed. The suggested CNF matches were then listed in ascending order of this maximal nutrition difference for each Euromonitor product. The first CNF item in this order was that with the smallest nutritional error. Figure 4 provides an example of how the most accurate match for a Euromonitor product with multiple algorithm-suggested matches was chosen.

Selection of the most accurate match was performed by a team of dietetics experts and registered dietitians. Two team members with dietetics expertise independently worked with the same set of algorithm-proposed matches. Any discrepancies in their final match selections were reviewed and decided by a third team member (registered dietitian). Any disagreement with the registered dietitian’s final selection was resolved as a full team.

#### 2.2.3. Manual match selection

Manual selection was conducted for Euromonitor products if: (1) they were unable to be run through the algorithm, (2) the algorithm did not propose any matches, or (3) among algorithm-proposed matches, none were accurate. Just as in the algorithm-aided selection process, manual match selection also used the subcategory and brand name of each Euromonitor product, its subcategory definition, and the CNF food name(s).

To limit subjectivity as much as possible, two team members with dietetics expertise were assigned the same set of Euromonitor products for independent manual selection. Discrepancies in manual selection were assigned to a third team member with dietetics expertise, who then also independently chose the best CNF equivalent. Then, one of our team’s registered dietitians reviewed the CNF matches suggested by those three team members and picked the best equivalent (which could also be a CNF food other than one of those suggested by the three colleagues). This final decision and its reasoning were reviewed together by all four of these individuals, and any lingering disagreement was discussed by the whole team until consensus was achieved.

### 2.3. Analyses

#### 2.3.1. Intercategories

For the purposes of reporting results, we generated a new level of categorization by collapsing multiple Euromonitor subcategories into so-called “intercategories.” This was necessary due to Euromonitor’s lack of a category level that would allow for dietetically meaningful reporting of results; 210 subcategories were far too many, while the 2 categories of study were too few. Each intercategory is composed of one or multiple subgroups of Euromonitor products, as indicated in Table 2. The intercategories are: Baby Food; Dairy; Ready Meals and Soup; Sauces, Dressings, Spreads, and Dips; Sweet Snacks; Savory Snacks; Baked Goods; Cereal and Grain Products; Processed Fruit and Vegetables; Processed Meat; Meat Substitutes; Processed Seafood; Soft Drinks and Juice; Coffee and Tea; and Water and Functional Beverages.

**TABLE 2:** The 15 intercategories generated for this work, the number of products in each intercategory (N), and the contributing Euromonitor subgroup levels (and their numbers of products = *n*).

Euromonitor subgroup level 1 (*n*)	Euromonitor subgroup level 2 (*n*)	Euromonitor subgroup level 3 (*n*)	Intercategory	Total number of products (*N*)
Dairy products and alternatives (180)	Baby food (20)	—	Baby food	20
	Dairy (160)	—	Dairy	160
Cooking ingredients and meals (220)	Ready meals (42)	—	Ready meals and soup	66
	Soup (24)	—		
	Sauces, dressings, and condiments (130)	—	Sauces, dressings, spreads, and dips	154
	Sweet spreads (24)	—		
Snacks (404)	Confectionery (181)	—	Sweet snacks	324
	Ice cream and frozen desserts (65)	—		
	Sweet biscuits, snack bars, and fruit snacks (78)			
	Savory snacks (80)	—	Savory snacks	80
Staple foods (213)	Baked goods (46)	—	Baked goods	46
	Breakfast cereals (30)	—	Cereal and grain products	56
	Rice, pasta, and noodles (26)	—		
	Processed fruit and vegetables (45)	—	Processed fruit and vegetables	45
	Processed meat and seafood (66)	Processed meat (38)	Processed meat	38
		Meat substitutes (9)	Meat substitutes	9
		Processed seafood (19)	Processed seafood	19
Carbonates (55)	—	—	Soft drinks and juice	126
Concentrates (17)	—	—		
Energy drinks (12)	—	—		
Juice (37)	—	—		
Sports drinks (5)	—	—		
Ready-to-drink coffee (7)	—	—	Coffee and tea	23
Ready-to-drink tea (16)	—	—		
Bottled water (13)	—	—	Water and functional beverages	13
			Total	1,179

#### 2.3.2. Descriptive statistics

Matching accuracy (overall and by intercategory) was measured as the number of Euromonitor products with an appropriate CNF match–from the algorithm and, separately, from both the algorithm and manual matching–divided by the total number of Euromonitor products of focus. For instance, the overall accuracy of the algorithm equaled the number of Euromonitor products that had at least one accurate algorithm-proposed match divided by the total number of Euromonitor products with at least one algorithm-proposed match.

Inter-rater agreement rates were calculated as the percentage of Euromonitor products for which both team members agreed on what to select or do. This was done for the algorithm-only part of this work (for agreement in selecting the same CNF match among algorithm options or refusing them) as well as for manual selection (agreement in selecting the same CNF equivalent).

## 3. Results

**FIGURE 5:**
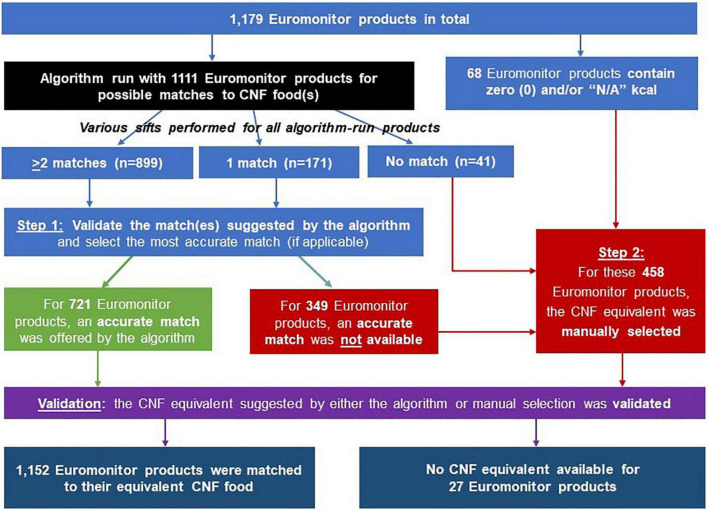
Flow diagram summarizing how many Euromonitor products were matched to their most accurate Canadian Nutrient File (CNF) equivalent *via* the algorithm-based and manual selection processes.

The flow diagram in Figure 5 summarizes the number of Euromonitor products that entered and matches that resulted from each step of our procedure. At the start, the Euromonitor data subset contained 1,179 branded products. Sixty-eight of these were identified as having zero calories or missing key nutrient information and were thus sent directly for manual matching.

**FIGURE 6:**
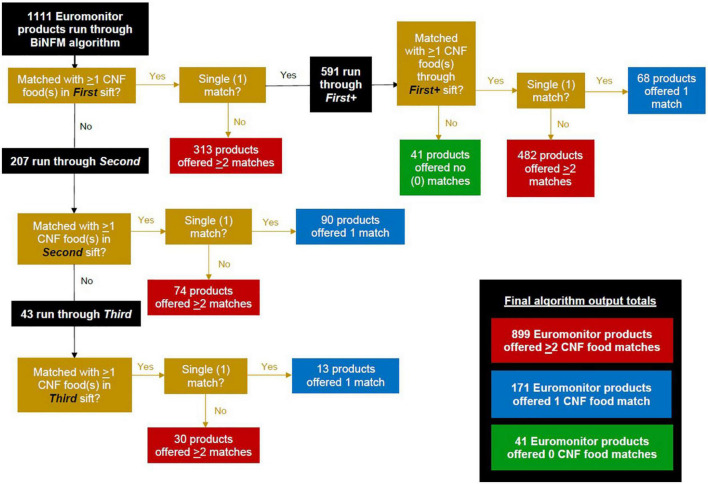
Overview of Euromonitor products’ coursing through the algorithm’s four sifts (by level of match output: 0, 1, or ≥2 matches).

In total, 1,111 Euromonitor products were run through our BiNFM algorithm, with 1,070 (96%) resulting in one or more algorithm-proposed matches. Figure 6 serves as a visual aid about the process of how, through each sift, three levels of matching were possible for each Euromonitor product: zero/no algorithm-proposed CNF match (= 0), a single/one match (= 1), or multiple matches (≥2). The “*First”* sift left 207 Euromonitor products without any potential CNF matches. The *“Second”* sift was applied with looser thresholds to these unmatched Euromonitor products, resulting in 43 unmatched products. Finally, these unmatched products from *“Second”* were run through “*Third*,” which provided suggested matches for all 43 products. Additionally, 591 Euromonitor products were sent through “*First*+.” At the end of the four sifts, 899 out of 1,111 Euromonitor products (81%) had been matched with two or more CNF foods; 171 (15%) of them matched with a single one; and 41 (4%) of them matched with none. All 41 of the ultimately unmatched Euromonitor products originally had a single CNF match in “*First”* and thus had entered “*First*+,” after which they became unmatched because their fuzzy match scores did not meet the algorithm’s threshold of 50 used in this latter sift. These by-sift numbers can also be found near the bottom of Table 3, along with the accuracy of algorithmic output for products with one (50%) or two or more (71%) match suggestions.

**TABLE 3:** Number of Euromonitor products run through the algorithm and the number and percentage of products accurately matched overall (total and by intercategory). Additionally, by intercategory and by level of algorithm-suggested matches, the numbers and percentages of proposed matches and of accurate such matches.

Intercategory	Number of products run in algorithm	Number and percent (%) of products accurately matched	Level of algorithm-proposed matches	Number and percent (%) of algorithm-proposed matches across the levels	Number and percent (%) of accurate algorithm-proposed matches by level
Baby food	20	10 (50.0)	0	0 (0.0)	N/A
			1	0 (0.0)	N/A
			≥2	20 (100.0)	10 (50.0)
Baked goods	45	25 (55.6)	0	0 (0.0)	N/A
			1	3 (6.7)	2 (66.7)
			≥2	42 (93.3)	23 (54.8)
Cereal and grain products	55	39 (70.9)	0	1 (1.8)	0 (0.0)
			1	7 (12.7)	0 (0.0)
			≥2	47 (85.5)	39 (83.0)
Coffee and tea	20	8 (40.0)	0	2 (10.0)	0 (0.0)
			1	6 (30.0)	4 (66.7)
			≥2	12 (60.0)	4 (33.3)
Dairy	157	127 (80.9)	0	6 (3.8)	0 (0.0)
			1	16 (10.2)	8 (50.0)
			≥2	135 (86.0)	119 (88.2)
Meat substitutes	9	8 (88.9)	0	0 (0.0)	N/A
			1	4 (44.4)	3 (75.0)
			≥2	5 (55.6)	5 (100.0)
Ready meals and soup	66	51 (77.3)	0	0 (0.0)	N/A
			1	4 (6.1)	3 (75.0)
			≥2	62 (93.9)	48 (77.4)
Processed fruit and vegetables	45	38 (84.4)	0	1 (2.2)	0 (0.0)
			1	2 (4.4)	0 (0.0)
			≥2	42 (93.3)	38 (90.5)
Processed meat	38	22 (57.9)	0	1 (2.6)	0 (0.0)
			1	0 (0.0)	N/A
			≥2	37 (97.4)	22 (59.5)
Processed seafood	19	8 (42.1)	0	8 (42.1)	0 (0.0)
			1	2 (10.5)	2 (100.0)
			≥2	9 (47.4)	6 (66.7)
Sauces, dressings, spreads, and dips	145	103 (71.0)	0	4 (2.8)	0 (0.0)
			1	32 (22.1)	23 (71.9)
			≥2	109 (75.2)	80 (73.4)
Savory snacks	80	57 (71.3)	0	1 (1.3)	0 (0.0)
			1	5 (6.3)	2 (40.0)
			≥2	74 (92.5)	55 (74.3)
Soft drinks and juice	96	61 (63.5)	0	2 (2.1)	0 (0.0)
			1	22 (22.9)	17 (77.3)
			≥2	72 (75.0)	44 (61.1)
Sweet snacks	313	164 (52.4)	0	15 (4.8)	0 (0.0)
			1	65 (20.8)	22 (33.9)
			≥2	233 (74.4)	142 (60.9)
Water and functional beverages	3	0 (0.0)	0	0 (0.0)	N/A
			1	3 (100.0)	0 (0.0)
			≥2	0 (0.0)	N/A
Total	1,111	721 (64.9%)	0	41 (3.7%)	N/A
			1	171 (15.4%)	86 (50.3%)
			≥2	899 (80.9%)	635 (70.6%)

Table excludes the 68 Euromonitor products sent directly to manual matching prior to the algorithm being run. Due to rounding, percentage totals may not total 100%.

**FIGURE 7:**
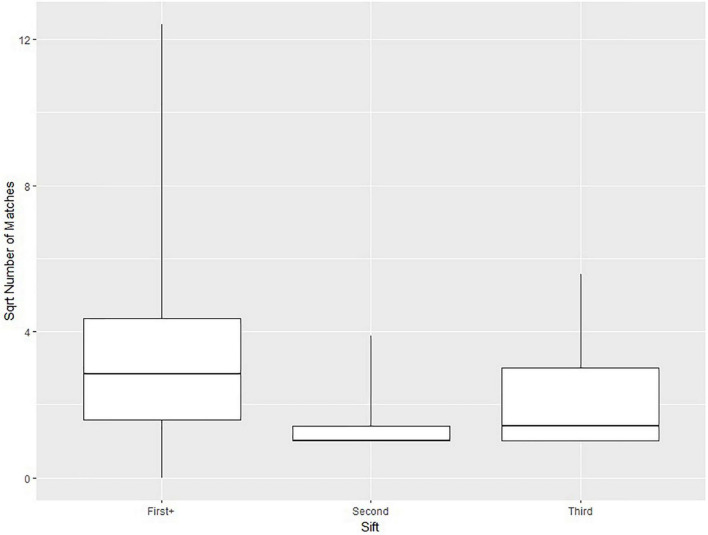
Boxplots indicating the 0th, 25th, 50th, 75th, and 100th percentiles of the square root of the number of matches across 3 of the sifts: “*First*+”, “*Second*”, and “*Third*”.

Figure 7 displays boxplots for the square root of the number of algorithmically suggested CNF matches for the Euromonitor products run in each of the sifts “*First*+” (591 products), “*Second”* (207 products), and “*Third”* (43 products). “*First”* is not included because every product in this sift had precisely one suggested CNF match. Square roots of the number of suggested matches are used instead of the raw numbers in order to make the plot more visually comprehensible but bears no other importance. Moving from “*First”* (excluded from the figure but equal to 1) to “*Second”* to “*Third”* sees increasing numbers of suggested matches. “*First*+” has the most suggested matches of all sifts.

Table 3 reports–by intercategory–the total number of Euromonitor products run in the algorithm and the number and percent of products accurately matched to an algorithm-suggested match. The following intercategories saw the highest percentage of products with an accurate algorithm-proposed CNF match: Meat Substitutes (89%), Processed Fruit and Vegetables (84%), and Dairy (81%). By contrast, Water and Functional Beverages (0%), Coffee and Tea (40%), and Processed Seafood (42%) had the lowest percentages after being run through the algorithm. Overall, of the 1,111 Euromonitor products that entered the algorithm, 721 (65%) resulted in a CNF match being selected among the algorithm suggestions.

That same table further breaks down, within each intercategory by level of algorithm matches (0, 1, or ≥2), how many algorithm-suggested matches there were, and how many of those were accurate. Out of the 15 intercategories, 13 (87%) saw the majority of their Euromonitor products end up with ≥2 algorithm-suggested CNF matches, with Processed Seafood just under half (47%). Water and Functional Beverages was the only intercategory with neither a plurality nor a majority of its products being offered ≥2 matches; instead, each of its 3 products in this intercategory had 1 algorithm-suggested CNF match. At levels 1 and ≥2, a majority of products had an accurate algorithm-proposed match, with higher accuracy observed for the latter: 50% of the 171 with 1 match option versus 71% of the 899 with ≥2 matches. The highest accuracies were observed among the following intercategories, with all at the ≥2 match level: Meat Substitutes (100%), Processed Fruit and Vegetables (90%), Dairy (88%), and Cereal and Grain Products (83%). Water and Functional Beverages was the only intercategory with 0% accuracy, as none of the single matches offered by the algorithm for its 3 products were nutritionally appropriate.

Out of the 1,179 total Euromonitor products, 1,152 (98%) were matched with a CNF equivalent either with an algorithm-suggested match or by manual selection in the CNF (Table 4). The exceptions were 3 products in Coffee and Tea (87%) and 24 in Sweet Snacks (93%); all 27 of these unmatchable products were a result of there being no nutritionally appropriate CNF match.

**TABLE 4:** Process accuracy, overall and by intercategory.

Intercategory	Number of products	Number of products after algorithm-based AND manual selection with an accurate CNF match	Number of products deemed unmatchable to CNF	Overall process accuracy as number matched (%)
Baby food	20	20	0	20 (100.0)
Baked goods	46	46	0	46 (100.0)
Cereal and grain products	56	56	0	56 (100.0)
Coffee and tea	23	20	3	20 (87.0)
Dairy	160	160	0	160 (100.0)
Meat substitutes	9	9	0	9 (100.0)
Ready meals and soup	66	66	0	66 (100.0)
Processed fruit and vegetables	45	45	0	45 (100.0)
Processed meat	38	38	0	38 (100.0)
Processed seafood	19	19	0	19 (100.0)
Sauces, dressings, spreads, and dips	154	154	0	154 (100.0)
Savory snacks	80	80	0	80 (100.0)
Soft drinks and juice	126	126	0	126 (100.0)
Sweet snacks	324	300	24	300 (92.6)
Water and functional beverages	13	13	0	13 (100.0)
Total	1179	1152	27	1152 (97.7%)

Inter-rater agreement rates by intercategory for both parts of the matching process–algorithm-based and manual–are reported in Table 5. The overall inter-rater agreement rate in the first step (selecting the same CNF equivalent among algorithm-suggested options or refusing all of those options) was 51%; the highest rates were in Water and Functional Beverages (100%) and Coffee and Tea (70%), while the lowest were seen for Processed Meat and Processed Seafood (both 32%). In terms of refusal of algorithm-proposed matches, the inter-rater agreement rate was 71% overall; this was highest among products in Baby Food and Water and Functional Beverages (both 100%) and lowest for those in Processed Meat and Processed Seafood (both 32%). The highest rate of agreement for algorithm-based selection was for Water and Functional Beverages (100%), while the lowest was among Processed Fruit and Vegetables (22%). For 5 of the 15 intercategories (31%), team members were more likely to agree to refuse the algorithm’s options–thus, sending those products to manual selection–than they were to agree on a specific algorithm-proposed option: Baby Food (100 vs. 65%); Dairy (85 vs. 43%); Ready Meals and Soup (86 vs. 58%); Sauces, Dressings, Spreads, and Dips (98 vs. 69%); and Sweet Snacks (81 vs. 54%). In the remaining 10 intercategories, those two agreement rates were equivalent (refusal of versus selection among algorithm-suggested options). Among the 407 Euromonitor products ultimately managed with manual selection, the overall inter-rater agreement rate of selecting the same CNF match was 33%.

**TABLE 5:** Inter-rater agreement rates in the algorithm-based and manual selection processes.

Intercategory	Algorithm-based selection			Manual selection	
	Number of Euromonitor products run through algorithm	Inter-rater agreement rate of selecting same CNF food or refusing algorithm option(s) (%)	Inter-rater agreement rate of deciding that manual selection is needed (%)	Number of Euromonitor products manually managed	Inter-rater agreement rate of selecting the same CNF equivalent (%)
Baby food	20	65.0	100.0	12	0.0
Baked goods	45	35.6	35.6	2	50.0
Cereal and grain products	55	65.5	65.5	8	12.5
Coffee and tea	20	70.0	70.0	12	50.0
Dairy	157	42.7	84.7	57	10.5
Meat substitutes	9	33.3	33.3	1	0.0
Ready meals and soup	66	57.6	86.4	49	34.7
Processed fruit and vegetables	45	22.2	22.2	1	100.0
Processed meat	38	31.6	31.6	2	100.0
Processed seafood	19	31.6	31.6	0	—
Sauces, dressings, spreads, and dips	145	69.0	97.9	139	29.5
Savory snacks	80	53.8	53.8	6	50.0
Soft drinks and juice	96	39.6	39.6	45	57.8
Sweet snacks	313	54.3	80.8	60	35.0
Water and functional beverages	3	100.0	100.0	13	76.9
Total	1,111	51.2%	70.8%	407	33.2%

## 4. Discussion

We developed, implemented, and documented an algorithm-assisted, expert-validated database mapping of Euromonitor Passport Nutrition’s branded food and beverage products sold in Canada between 2014 and 2018 to their respective equivalents in the national food composition database, the CNF. The use of an algorithm helped optimize the efficiency of an otherwise fully manual initiative–saving time and labor. Our algorithm design is readily applicable to other contexts, as the parameters from the Euromonitor and CNF databases that we utilized are not unique in the food-related research arena. The two core requirements are a text descriptor of a food or product (for fuzzy matching) and some nutrient data; nearly all such datasets possess the former, with many also containing the latter. The use of a third food categorization system in common (the BNS) is an optional asset to further focus the algorithm’s database search (in our case, of the CNF). Our approach to nutrient threshold selection combined numerical heuristics with expert judgment calls; however, one could just as well employ other parameter or threshold selection techniques to suit their needs and problem context. It is important to remark that there are two processes presented in this report. One process is the algorithm for producing suggested matches; the other is the flow of the various sifts. Multiple sifts were used because, while some Euromonitor items had nutritionally appropriate CNF match suggestions in our initial sift (“*First”*), other items did not have ideal matches. Therefore, we wanted to keep the matches that were potentially good in our first run of the algorithm, but then re-run the algorithm with different sets of parameters to obtain alternative suggested matches for those Euromonitor products with poor or no suggested matches in the previous run. The integration of dietetics expertise to validate our CNF match choices ensured that these selections were appropriate based on products’ nutrition information and subcategory definitions, the latter of which the algorithm was unable to leverage. Thus, despite the time and labor it added to the process, dietetics expertise was an imperative supplement to the algorithm-based matching effort, as the rigor of our planned future studies using this CNF-linked Euromonitor dataset depends on the precision of this database mapping.

In the end, 1,152 (98%) of the Euromonitor products matched with a CNF food, with the remaining 27 (2%) unmatchable products owing to a lack of an equivalent food available in the CNF. All products from the following Euromonitor subcategories were unmatchable: Lollipops, Medicated Confectionery, Power Mints, Fruit and Nut Bars, and Carbonated Ready-To-Drink Tea. Brinkerhoff et al. similarly tracked reasons for unmatchability in their manual matching effort of food sold at a supermarket, and they found 4.6% of food products were not covered by the USDA-SR (4).

Like other examples in the literature, we sought to design our algorithm in a way that would maximize both the overall quality and accuracy of matches (11, 12). We also wanted the algorithm to provide at least one match suggestion for each Euromonitor product, which we were able to achieve for nearly all products. The only reason 41 products were left without a match was due to the “*First*+” sift. In our effort to raise the number of algorithm suggestions from a single match option in *“First,”* the addition of fuzzy matching with a threshold of 50 in “*First*+” may have been too stringent. Of the 591 products run through *“First*+,” 41 (7%) of these products failed to meet this fuzzy match threshold and were ultimately left with no CNF match, as we did not retain the single match option from *“First”* (which instead had used fuzzy match optimization rather than a strict fuzzy matching threshold). While increasing the fuzzy matching threshold would likely have added more options to wade through (particularly for those 41 without any algorithm options), this would also have reduced the overall sensitivity of *“First*+.”

The fewer the number of Euromonitor products needing manual selection after being run through the algorithm, the higher the algorithm’s accuracy. As anticipated, those products with multiple algorithm suggestions had higher match accuracy versus those with only one suggestion (71 vs. 50%). Future algorithms could require multiple matches, but, like with the fuzzy matching loosening, this would then increase the possibility that thresholds would become too loose. This would render the algorithm less sensitive and increase the resource burden of choosing between multiple match options for a greater proportion of the products–costing labor and time while lowering process efficiency overall.

Compared to similar published endeavors, we find that our matching experience was resonant in some ways and distinct in others. Like Thiele et al., who linked foods in GfK to their equivalents in the German food composition database, we also found that several Euromonitor products could be linked to the same (usually generic) CNF food. However, unlike their team, ours did not find the sales database possessed “extremely in-depth documentation” on food composition relative to the national food composition database (16). The semi-automated approach of Carter et al. (17) is akin in certain ways to our matching algorithm, but with some notable differences; most importantly, they appear to compare the percent difference in nutrients rather than the differences in the proportions of calories per nutrient as we have done. As we have posited, using simple percent differences is a less robust way of comparing the nutrients for low-calorie foods, and so one might expect that Carter et al.’s algorithm could have encountered issues matching these items. Another important difference with their work is our added use of fuzzy string matching to further aid our sifts in identifying the best possible algorithm-suggested matches in the CNF (17).

While this field is pushing further into fully automated approaches like artificial intelligence and natural language processing, dietetics expertise remains critically invaluable for many database mapping endeavors (18, 19). This is particularly true for datasets where the context of nutrients, food categorization systems (e.g., too-vague or too-detailed), and other heuristic aspects of matching are not easy for a computer to handle. Algorithms like ours therefore offer a pragmatic way to aid the matching process yet are not intended as a one-size-fits-all, complete solution to such matching problems. In their largely automated approach to food database mapping, Bohn et al. (20) observed that fuzzy string matching was inhibited by the non-standardized naming of food in producers’ databases and had an expert manually check low-similarity potential matches. We, too, experienced this naming quandary in Euromonitor, which we also addressed with manual effort by dietetics experts. Additionally in our case, because Euromonitor is often used for market research data, its subcategories were sometimes named for marketing and retail purposes; as a result, definitions were necessary to be used in conjunction with Euromonitor subcategory names to fully understand the products within them. For example, the subcategory “Countlines” is defined as “chocolate bars eaten as snacks,” which was critical added information for matching. In future work, an expert understanding of food-related database architecture and terminologies could be used to develop appropriate text-based fuzzy strings to add to the matching algorithm without sacrificing sensitivity.

Unlike algorithm-only approaches that leverage fuzzy (or other automated text-based) matching approaches–and more akin to fully manual matching efforts–we wanted to ensure that match accuracy was not merely based on the closeness in matched food names, but that the food composition would be as nutritionally close as possible, too. Unfortunately, as previously noted, food is largely unstandardized in its terminology. This is likely owed to their distinct purposes: Euromonitor for market analyses versus CNF for federal health survey analyses. Thanks to Euromonitor and CNF entries both having data for key nutritional variables, we were able to dietetically validate final match selections using both calculated nutrient differences and food names (and, if necessary, Euromonitor subcategory definitions and brand names).

Dietetics expertise therefore played a vital role in this endeavor and was a core strength of our methodology. Instructions for validating the algorithm’s proposed matches and the manual selection process were developed by team leads with extensive knowledge and clinical dietetic experience relevant to food composition, the Canadian food supply, and the implications of nutrition on health outcomes. We were able to minimize subjectivity by training team members to follow a detailed matching protocol. Other major strengths of our methodological contribution to the discipline include the low-bias and longitudinal nature of the Euromonitor dataset for Canada. We also were able to partially solve the problem faced by Lamarine et al. of nutrient variability, or “variability between different versions of the same food item. For example, 100 g portion of raw garlic would be recorded with an energy content varying between 305 and 670 kcal” (12). While they argued “data curation (including detection and correction of errors) remains a challenge and a thorough review of each composition variables cannot be performed without automated approaches,” we were fortunate to be able to innovate with and incorporate nutrient thresholds for matching in our algorithm, as Euromonitor had key nutritional data (12).

In terms of limitations, our algorithm design was restricted to those 7 nutrients and energy available in both databases; as such, the inclusion of fuzzy matching to draw on text-based data between the two databases proved to be a crucial addition. We were also unable to send products with missing nutrient data and/or zero calories into the algorithm, with the latter due to the non-sodium nutrient thresholds using energy as a denominator. The algorithm’s BNS food group restriction was helpful in achieving a more focused set of suggested matches. However, due to product heterogeneity within some Euromonitor subcategories, this may have disadvantaged the algorithm by potentially missing out on some CNF match options that may not have fallen precisely within the preselected BNS group; we found this to be a limited concern, almost exclusively and minimally affecting the following 3 Euromonitor subcategories: Ready Meals, Processed Meat, and Processed Seafood. Brinkerhoff et al. reported a similar issue when fully manually matching their subcategories (so-called “sub-commodities”) to the USDA-SR; they were unable to link 21% of them (“∼30% of the entire dataset”) due to “heterogeneous sub-commodities containing nutritionally diverse food items that could not be mapped to a single [USDA-]SR item entry” (4). There is also subjectivity inherent in the evaluation of database mapping, as the algorithm can only offer us choices; we must make the final selections. We attempted to mitigate risk of bias and human error by the rigor of and fidelity to our aforementioned, standardized, expert-led match selection at each step. While our inter-rater agreement rates were only 51% among algorithm suggestions and 33% among manual CNF selections, it is important to think about the nuanced, oft-small differences between very similar options in the CNF. Our team discovered it is harder to agree on the same “best” CNF equivalent than it is to refuse all algorithm options and simply assign that Euromonitor product to manual matching. This is evidenced by the fact that no inter-rater agreement rate for algorithm-based selection was higher than that for send-off to match selection (in other words, algorithm option refusal). Most intercategories’ rates were equal across these two sub-steps, with only 5 intercategories having a lower agreement rate among the former than the latter. This ties back to the value of nomenclature in a discipline, as we did not always have specific product names in the Euromonitor dataset. This is why we relied on a combination of all data at our disposal throughout the process: subcategory names (using fuzzy matching), BNS food groups (as a search restriction), and nutrient thresholds in the algorithm as well as subcategory definitions and brand names.

By choosing the nutrient thresholds we selected in our algorithm, we gave ourselves an upper bound on match quality. It only takes one nutrient beyond the threshold for the algorithm to reject a potential CNF match. In this sense, our approach was quite conservative. Multiple rounds of dietetic expert validation of the final match selection—with two independent validators plus a registered dietitian—ensured that branded products’ matches were nutritionally appropriate (per our stated goal). It is possible that some of the matches to the more generic CNF foods might not be best suited for those micronutrients for which we lacked data on the Euromonitor side (e.g., vitamin D content in a particular brand of a fortified breakfast cereal versus that in its generic match in the CNF). This possible source of nutritional discrepancy limits our potential use of these matched datasets for certain population nutrition studies, as we can only be confident for those 7 nutrients and energy data from Euromonitor and that we have been able to utilize and validate in this matching effort.

With the possible exception of the BNS food group bridging, the BiNFM algorithm is flexible enough to conceivably be applied to the matching of databases other than CNF and Euromonitor. The steps of the algorithmic model we developed can be immediately applied to datasets bearing the same kinds of nutritional data (e.g., energy, carbohydrates, proteins, total fat, fiber, saturated fat, sugar, and sodium) as well as some type of string to be fuzzy-matched. The BiNFM algorithm restricted matches to products with compatible BNS food groups, but this step can be omitted or replaced with restricting matches to compatible categories from another scheme. Even the list of nutrients could be changed, or string fuzzy matching could be omitted altogether. Importantly, the BiNFM algorithm relies heavily on products having non-zero energy content (a requirement for our computation of nutrient differences) and non-missing nutritional data.

## 5. Conclusion

To the best of our knowledge, this paper constitutes the first algorithm-aided matching of any marketing database’s branded food and beverage products sold in Canada to their nutritional equivalents in the CNF. As far as we are aware, this is also the first paper to detail the dietetic expert-driven validation of that matching process, which has now laid the groundwork for rigorous population nutrition and health research using the Euromonitor products’ nutrient profiles, sales, and other variables. Indeed, the linkage of food composition data to products found in marketing databases for public health nutrition studies is still a relatively nascent and emerging field, with much of the literature in this space published within the last 15 years. As food supply, retail, marketing, and other related databases become increasingly recognized as ripe opportunities for population nutrition surveillance, methods like ours can be used to enrich analyses of Euromonitor product trends (as the CNF matches offer additional nutrient data) and to supplement national health and dietary surveys with branded food composition data (available from the now-linked Euromonitor products). Although the specific parameters and architecture of our two datasets shaped the most granular details of our matching methodology, we are confident that the overall approach (including the algorithm design) that we employed and trade-offs we weighed would be generalizable and of assistance in similar food-matching endeavors.

## Data availability statement

The data analyzed in this study is subject to the following licenses/restrictions: The Passport Nutrition dataset for Canada that was used in this work was purchased from Euromonitor International, and so access follows regulations pertaining to it. Requests to access these datasets should be directed to MJ, mahsa.jessri@ubc.ca.

## Author contributions

SG contributed to the brainstorming and interpretation of the algorithm, analyzed both the algorithm-based and manual matching processes, led figure production, and led the writing of this manuscript. CM programmed and led development of the BiNFM matching algorithm, was involved in discussions of results, and participated in the writing of the manuscript and figure production. QC prepared and restructured the Euromonitor and Canadian Nutrient File datasets for the algorithm, assisted the development of the matching algorithm, co-led the algorithm validation and manual matching processes and analyses, and participated in the writing of the manuscript and figure production. JP conducted a literature review as background and assisted in drafting the manuscript. JB supervised the dietetic validation, manual matching processes, and provided editing support for the manuscript. MJ conceptualized and presented the idea of this work, and oversaw all activities related to this project, including algorithm design, execution of it as well as the manual matching process, manuscript preparation, and project/grant administration. All authors approved of the final version of this manuscript for publication.
